# Characterization of Apoptosis Induced by Emodin and Related Regulatory Mechanisms in Human Neuroblastoma Cells

**DOI:** 10.3390/ijms141020139

**Published:** 2013-10-09

**Authors:** Fu-Jen Huang, Yan-Der Hsuuw, Wen-Hsiung Chan

**Affiliations:** 1 Department of Obstetrics and Gynecology, Kaohsiung Chang Gung Memorial Hospital, Kaohsiung 83301, Taiwan; E-Mail: huangfj@seed.net.tw; 2 Chang Gung University College of Medicine, Kaohsiung 83301, Taiwan; 3 Department of Life Science, National Pingtung University of Science and Technology, Pingtung 91201, Taiwan; E-Mail: hsuuw@yahoo.com.tw; 4 Department of Bioscience Technology and Center for Nanotechnology, Chung Yuan Christian University, Chung Li 32023, Taiwan; 5 Institute of Biomedical Technology, Chung Yuan Christian University, Chung Li 32023, Taiwan

**Keywords:** emodin, apoptosis, oxidative stress, calcium, nitric oxide

## Abstract

Emodin (1,3,8-trihydroxy-6-methylanthraquinone), a major constituent of rhubarb, has a wide range of therapeutic applications. Recent studies have shown that emodin can induce or prevent cell apoptosis, although the precise molecular mechanisms underlying these effects are unknown. Experiments from the current study revealed that emodin (10–20 μM) induces apoptotic processes in the human neuroblastoma cell line, IMR-32, but exerts no injury effects at treatment doses below 10 μM. Treatment with emodin at concentrations of 10–20 μM led to a direct increase in the reactive oxygen species (ROS) content in IMR-32 cells, along with significant elevation of cytoplasmic free calcium and nitric oxide (NO) levels, loss of mitochondrial membrane potential (MMP), activation of caspases-9 and -3, and cell death. Pretreatment with nitric oxide (NO) scavengers suppressed the apoptotic biochemical changes induced by 20 μM emodin, and attenuated emodin-induced p53 and p21 expression involved in apoptotic signaling. Our results collectively indicate that emodin at concentrations of 10–20 μM triggers apoptosis of IMR-32 cells via a mechanism involving both ROS and NO. Based on the collective results, we propose a model for an emodin-triggered apoptotic signaling cascade that sequentially involves ROS, Ca^2+^, NO, p53, caspase-9 and caspase-3.

## 1. Introduction

Emodin (1,3,8-trihydroxy-6-methylanthraquinone), one of the major chemical constituents of rhubarb root (*Rheum palmatum* L.), is widely used in the Orient [1], and exerts immunosuppressive, anti-cancer, anti-inflammatory, anti-atherosclerotic, and vasorelaxant effects [2–5]. Emodin has been shown to inhibit proliferation in different cancer cell lines, including HER-2/neu-overexpressing breast cancer [6], hepatoma [7], leukemia [8], and lung cancer [9]. Moreover, emodin-stimulated apoptosis is mediated via reactive oxygen species (ROS) and mitochondria-dependent pathways in human tongue squamous cancer SCC-4 cells [10]. Interestingly, emodin appears to exert both cytotoxic and protective effects in rat C6 glioma cells [11]. Recent experiments by our group showed that emodin induces a decrease in mouse embryonic development and viability, both *in vitro* and *in vivo* [12]. Interestingly, the hazardous effects of emodin on embryonic development were effectively prevented by caspase-9 and -3 inhibitors [12], implying that emodin triggers impairment of blastocyst development through intrinsic apoptotic pathways. However, while the apoptotic effects of emodin have been validated, the precise underlying molecular mechanisms are yet be fully elucidated.

Numerous chemical and physical treatments capable of inducing apoptosis stimulate oxidative stress via ROS generation in cells [13–15], suggesting a close relationship between oxidative stress and apoptotic death. Nitric oxide (NO) is an important second messenger involved in a variety of cellular responses and biological functions, including tumor development, metastasis and apoptosis [16–18]. NO is predominantly produced in mitochondria through the actions of Ca^2+^-sensitive mitochondrial NO synthase (NOS) [19,20], and possibly regulates oxygen consumption and mitochondrial membrane potential through cytochrome c oxidase. The NO molecule is subsequently reactivated with superoxide to produce peroxynitrite, leading to further modification of its target substrates and induction of oxidative stress [21–25]. Oxidative stress and Ca^2+^ influx act as upstream regulators of mitochondrial NOS activity [26,27]. Another recent study showed that emodin triggers ROS and Ca^2+^ production in SCC4 cells [10], implying that ER stress and Ca^2+^ are involved in emodin-induced apoptotic processes.

To elucidate the precise regulatory mechanisms underlying emodin-triggered apoptosis in neuroblastoma cells, we examined the effects of emodin on the human cell line IMR-32. Based on the results obtained, a model of emodin-induced cell apoptotic signaling in IMR-32 cells is proposed.

## 2. Results

### 2.1 Cytotoxic Effects of Emodin on IMR-32 Cells

**Figure 1:**
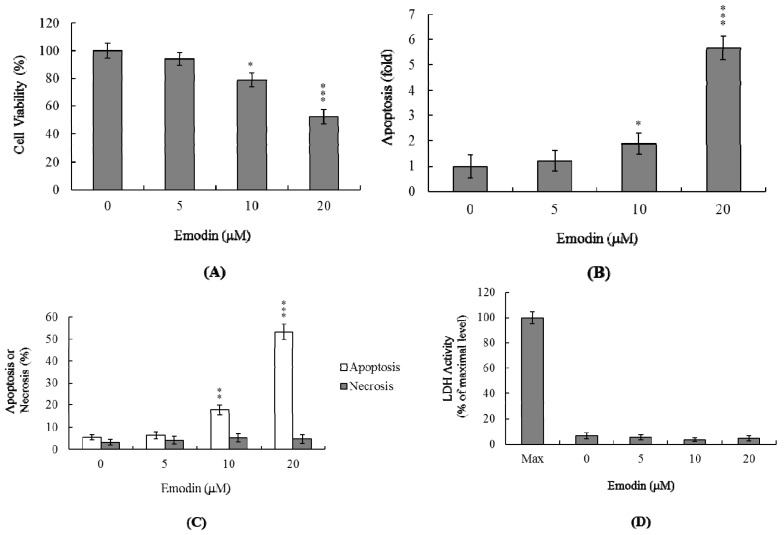
Effects of emodin on IMR-32 cells. IMR-32 cells were incubated with 0–20 μM emodin for 24 h. (**A**) Cell viability was determined using the MTT assay; (**B** and **C**) Apoptosis was detected with the Cell Death Detection ELISA kit (**B**); followed by staining with propidium iodide and Hoechst 33342 (**C**); (**D**) Necrosis was further assessed based on lactate dehydrogenase (LDH) activity release in the culture medium, and data expressed as a percentage of the maximal level (Max) of LDH activity determined after total cell lysis. Values are presented as means ± SD of eight determinations. * *p* < 0.05, ** *p* < 0.01 and *** *p* < 0.001 *versus* the untreated control group.

To assess the effects of emodin on IMR-32 cells, we employed the MTT assay to determine the viability of cells treated with various doses of the compound. Treatment concentrations of less than 10 μM had no effects on cell viability, while treatment with 10 μM emodin notably induced cell death compared to 5 μM (Figure 1A). A TUNEL ELISA kit was employed, with the aim of establishing the precise mode of emodin-induced cell death. Notably, 20 μM of emodin induced a 5.7-fold increase in TUNEL positivity, compared with untreated cells (Figure 1B). The percentages of apoptotic and necrotic cells were further evaluated by staining with propidium iodide and Hoechst 33342. As shown in Figure 1C, the proportion of apoptotic cells was significantly increased following treatment with 10–20 μM of emodin, while no necrotic cells were identified in the emodin-treated group (Figure 1C,D). Our findings collectively indicate that emodin induces apoptosis, and not necrosis, at concentrations greater than 10 μM in IMR-32 cells, but does not exert cytotoxic effects at doses lower than 10 μM.

### 2.2 ROS Levels Are Increased in IMR-32 Cells Treated with Emodin

**Figure 2:**
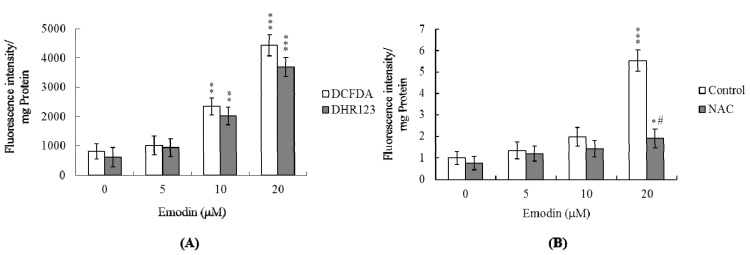
Emodin promotes oxidative stress in IMR-32 cells. (**A**) IMR-32 cells were treated with 0–20 μM emodin for 24 h, and ROS generation assayed using DCF-DA (20 μM) or dihydrorhodamine 123 (DHR 123; 20 μM); (**B**) IMR-32 cells were pre-incubated with *N*-acetyl cysteine (NAC; 300 μM) for 30 min, followed by treatment with or without emodin, as indicated. Reactive oxygen species (ROS) generation was assayed using DCF-DA. Data are representative of eight independent experiments. *p < 0.05, ** *p* < 0.01 and *** *p* < 0.001 *versus* the untreated control group. # *p* < 0.001 *versus* the emodin-treated group.

In view of our previous finding that numerous chemical stimuli trigger apoptosis via ROS generation [28,29], we employed DCF-DA and DHR 123 to examine ROS formation in IMR-32 cells treated with emodin. Emodin (10–20 μM) stimulated ROS generation about 2.8–5.4-fold, compared with the untreated control group (Figure 2A). Notably, pretreatment with *N*-acetyl cysteine (NAC), a commonly used ROS scavenger, effectively prevented ROS production in the presence of 10–20 μM of emodin (Figure 2B). Our results clearly indicate that the mechanism of emodin-induced apoptosis of IMR-32 cells involves ROS generation.

### 2.3 Changes in Intracellular Ca^2+^ and NO Levels Are Involved in Emodin-Induced Cell Apoptosis

**Figure 3:**
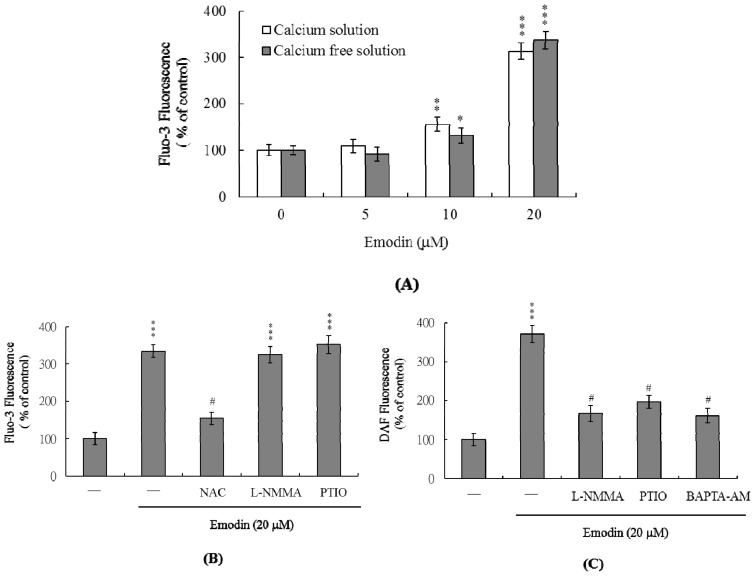
Emodin induces changes in the intracellular calcium and nitric oxide (NO) content of IMR-32 cells. (**A**) IMR-32 cells were incubated with 0–20 μM emodin for 24 h. Intracellular Fluo-3 fluorescence intensity was measured in the presence/absence of extracellular Ca^2+^; (**B**) Intracellular Ca^2+^ level changes following treatment with 20 μM emodin and effects of ROS and NO inhibitors (NAC: 300 μM; L-NMMA: 400 μM; PTIO: 20 μM) were examined; (**C**) IMR-32 cells were pretreated with L-NMMA (400 μM), PTIO (20 μM) or BAPTA-AM (10 μM) for 30 min, followed by incubation in the presence or absence of 20 μM emodin. Intracellular NO generation was measured using DAF-2DA fluorescence dye. Data are presented as a percentage of the control group. * *p* < 0.05, ** *p* < 0.01 and *** *p* < 0.001 *versus* the untreated control group. # *p* < 0.001 *versus* the “emodin-treated” group.

Changes in [Ca^2+^]i in emodin-treated IMR-32 cells were detected using Fluo-3AM fluorescence dye. Treatment with 10–20 μM emodin elicited an increase in [Ca^2+^]i (Figure 3A). Furthermore, cells cultured in Ca^2+^-containing medium displayed a ~3.1-fold increase in [Ca^2+^]i following treatment with 20 μM emodin, while those cultured in Ca^2+^-free medium showed no effect (Figure 3A). These findings indicate that the increase in [Ca^2+^]i is primarily attributed to release of internal Ca^2+^, similar to that observed in endoplasmic reticulum, mitochondria, nucleus and/or calcium-binding proteins (Figure 3A).

PTIO, an inhibitor of NOS and scavenger of NO, and L-NMMA, an inhibitor of NO synthase (NOS), had no effects on the [Ca^2+^]i increase induced by 20 μM emodin, whereas pretreatment with NAC significantly suppressed this increase (Figure 3B). Thus, it appears that elevation of [Ca^2+^]i by emodin is regulated by ROS, but not NO. We further used the NO-sensitive dye DAF-2DA to measure intracellular NO generation during emodin-induced apoptosis. Notably, intracellular NO levels were increased in IMR-32 cells treated with 20 μM emodin (Figure 3C). This increase was prevented upon pretreatment of cells with the NOS inhibitor, L-NMMA and PTIO, or 10 μM BAPTA-AM (a Ca^2+^ chelator) (Figure 3C). Based on these findings, we propose that intracellular Ca^2+^ levels play an important role in NOS activation and NO increase observed in emodin-treated IMR-32 cells.

### 2.4 PTIO Inhibits Mitochondrial Membrane Potential (MMP) Changes and Caspase Activation during Emodin-Induced Cell Apoptosis

**Figure 4:**
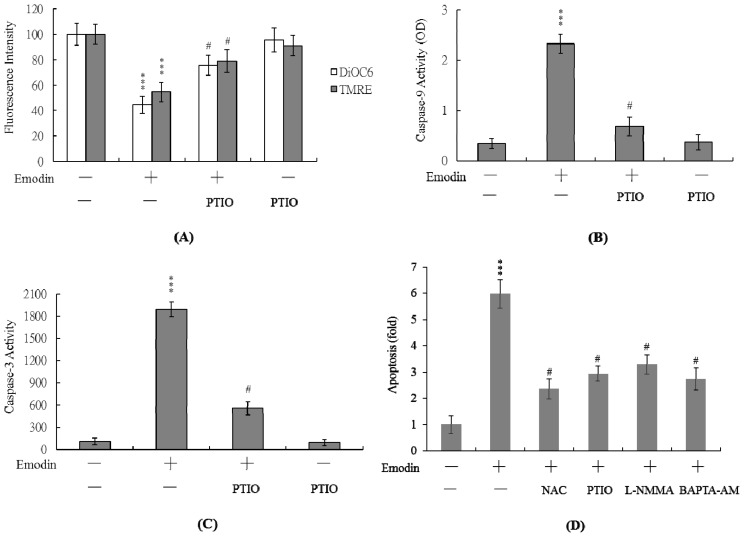
MMP alterations and activation of caspases-9 and -3 following emodin treatment of IMR-32 cells. IMR-32 cells were pretreated with PTIO (20 μM) for 1 h, and treated with 20 μM emodin or left untreated for a further 24 h. (**A**) Mitochondrial membrane potential changes were analyzed using 40 nM DiOC6(3) or 1 μM TMRE; (**B**) Caspase-9 activity was assayed using the Colorimetric Caspase-9 Assay kit; (**C**) Cell extracts (60 μg) were analyzed for caspase-3 activity using Z-DEVD-AFC as the substrate; (**D**) IMR-32 cells were pretreated with NAC (300 μM), PTIO (20 μM), L-NMMA (400 μM) or BAPTA-AM (10 μM) for 1 h, and treated with 20 μM emodin or left untreated for a further 24 h. Apoptosis was detected with the Cell Death Detection ELISA kit. *** *p* < 0.001 *versus* the untreated control group. # *p* < 0.001 *versus* the “emodin-treated” group.

Next, we analyzed changes in MMP, a major apoptotic event during mitochondrial-mediated apoptosis. Uptake of DiOC6(3) and TMRE into mitochondria of IMR-32 cells was observed, indicating significant loss of MMP following treatment with 20 μM emodin (Figure 4A). In addition, we monitored activation of caspases-9 and -3 involved in mitochondria-mediated apoptotic pathways. Treatment of IMR-32 cells with 20 μM emodin clearly stimulated caspase-9 (Figure 4B) and -3 (Figure 4C) activities. Importantly, both MMP loss and caspase activation were significantly inhibited upon incubation of cells with 20 μM PTIO, prior to co-treatment with 20 μM emodin (Figure 4A–C). Moreover, we found that pretreatment with NAC, PTIO, L-NMMA or BAPTA-AM effectively blocked emodin-triggered apoptosis in IMR-32 cells (Figure 4D). These results indicate that ROS, NO and Ca^2+^ act as upstream regulators of MMP changes and activation of caspases-9 and -3 during emodin-induced apoptosis.

### 2.5. Changes in p53 and p21 Expression Levels Following Emodin Treatment of IMR-32 Cells

**Figure 5:**
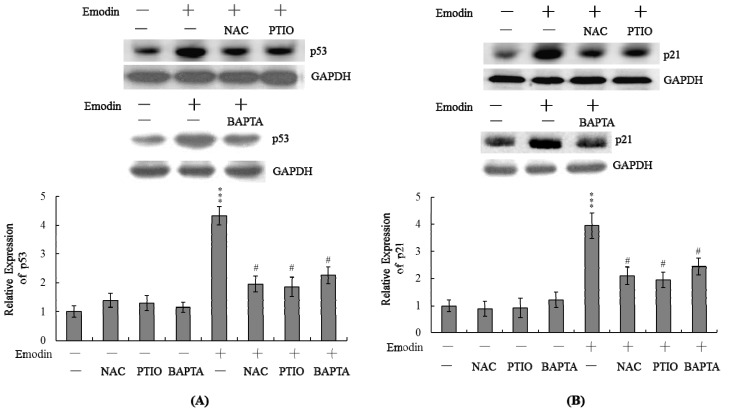

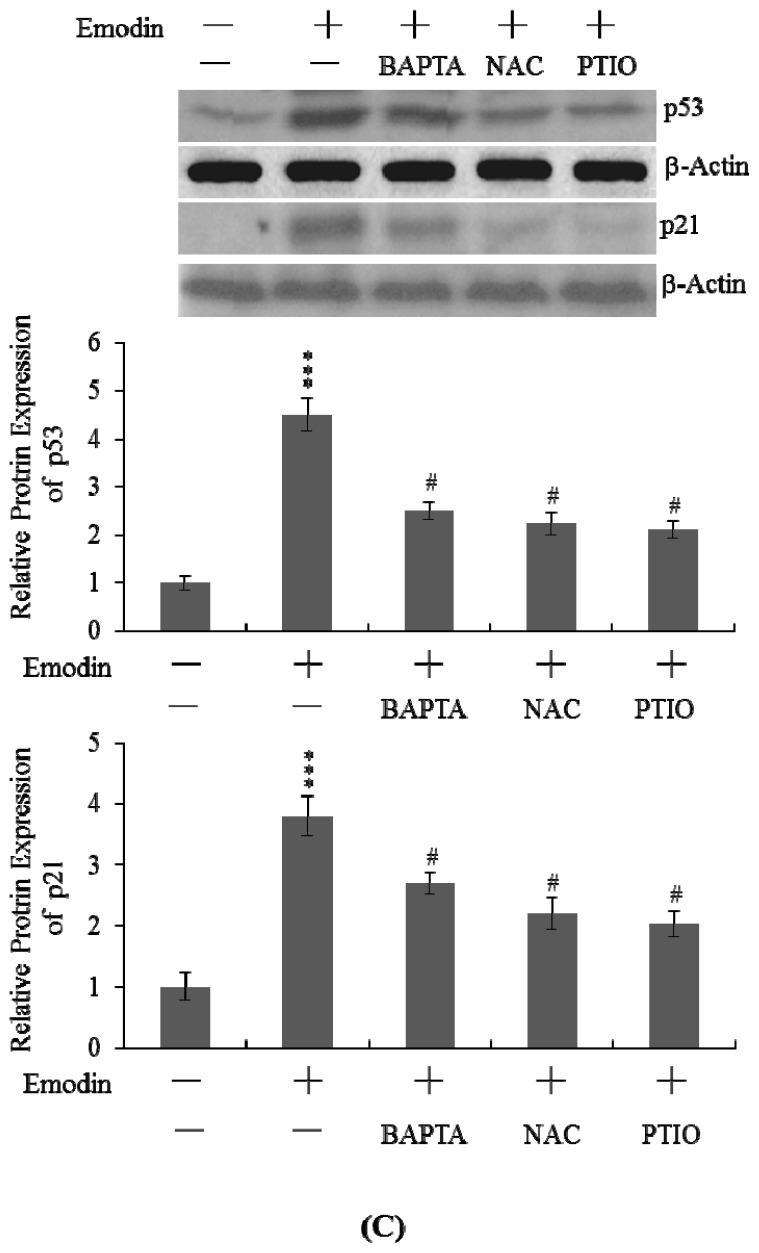
Effects of NAC and PTIO on p53 and p21 mRNA levels. IMR-32 cells were either pre-incubated with NAC (300 μM), PTIO (20 μM) or BAPTA-AM (BAPTA; 10 μM) for 1 h or left untreated, followed by treatment with 20 μM emodin for another 24 h. The mRNA levels of p53 (**A**) and p21 (**B**) were analyzed using real-time PCR. Quantification and agarose gel electrophoresis data of p53 (**A**) and p21 (**B**) were analyzed using real-time PCR; (**C**) Protein expressions of p53 and p21 were detected by immunoblotting. Values are representative of eight determinations. *** *p* < 0.001 *versus* the untreated control group. # *p* < 0.001 *versus* the “emodin-treated” group.

A previous study found that pretreatment of human hepatoma HepG2 cells with aloe-emodin, isolated from the rhizomes of Rheum palmatum, could cause cell cycle arrest at G1-phase. Aloe-emodin regulation of the cell cycle was found to be associated with up-regulation of p53 and p21 [30]. In addition, NO-mediated apoptotic processes are associated with p53 gene activation, which is essential for regulation of the cell cycle and/or apoptotic signaling occurring through p21^Waf1/Cip1^ or Bax [31,32]. These findings prompted us to further investigate where p53 and p21 are involved in emodin-induced cell apoptotic processes. Data from real-time RT-PCR and immunoblotting analyses revealed significant upregulation of p53 and p21 mRNA and protein expression in IMR-32 cells treated with 20 μM emodin, which was blocked upon pretreatment with NAC, PTIO or BAPTA-AM (Figure 5A–C).

### 2.6. Treatment of IMR-32 Cells with p53 siRNA Blocks Emodin-Induced Apoptosis

**Figure 6:**
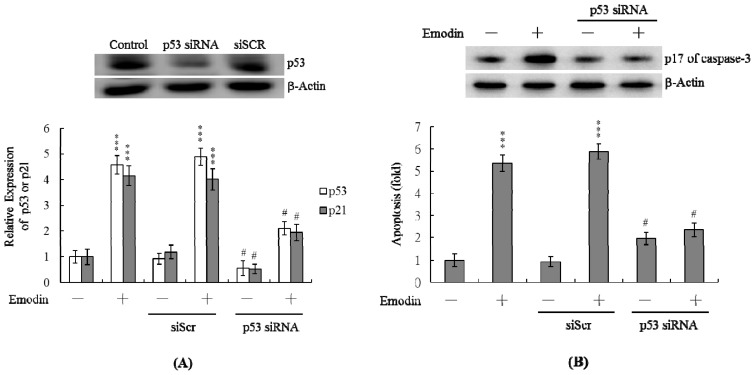
Knockdown of p53 protects IMR-32 cells against emodin-induced apoptosis. IMR-32 cells were transfected with siRNA targeting p53 and scrambled siRNA duplex (siScr), incubated for 24 h, and treated with 20 μM emodin for a further 24 h. (**A**) The protein expression levels of p53 were detected by immunoblotting. The mRNA levels of p53 and p21 were analyzed using real-time PCR; (**B**) Active form caspase-3 (cleavage products of caspase-3; p17) were detected by immunoblotting. Apoptosis was detected with the Cell Death Detection ELISA kit. *** *p* < 0.001 *versus* the untreated control group. # *p* < 0.001 *versus* the “emodin-treated” group.

To further establish the roles of p53 and p21 in emodin-induced apoptosis, we used targeted siRNAs to suppress p53 expression in IMR-32 cells. We transfected IMR-32 cells with scrambled siRNA (siScr) and found that the expression levels of p53 did not significantly differ between the siScr-transfected and un-transfected control groups. Cells were transfected with *p53 siRNA* could effectively suppress p53 protein expression (Figure 6A). Cells were incubated with 20 μM emodin for 24 h, and subsequently tested for viability. Knockdown of p53 led to a significant decrease in p53 and p21 mRNA levels in emodin-treated IMR-32 cells (Figure 6A), accompanied by marked suppression of emodin-induced apoptosis (Figure 6B). Our results indicate that emodin-induced upregulation of p53 and p21 in IMR-32 cells subsequently promotes apoptosis.

## 3. Discussion

Emodin, a natural chemical compound present in the root of rhubarb, is widely used in Chinese medicine. Recently, Wei and colleagues demonstrated that emodin not only induces apoptosis of cancer cells directly but also enhances the anti-cancer effects of gemcitabine in pancreatic cancer [33]. Treatment with gemcitabine in combination with emodin efficiently inhibited tumor growth in mice inoculated with pancreatic tumor cells. This combination therapy induced a reduction in Akt and NF-κB activation and the Bcl-2/Bax ratio, and increased caspase-9 and -3 activation, as well as cytochrome C release from mitochondria to cytosol [33]. Thus, emodin appears to function as a chemopreventive and/or chemotherapeutic agent in different cancer types by decreasing cell viability, inhibiting cell proliferation, and increasing apoptosis. Oxidative stress is a stimulator of several cell responses, including apoptosis [34,35]. A previous study showed that emodin acts as a ROS generator to trigger cell death in tumor cells, supporting its utility as a cytotoxic therapeutic agent [9]. Another investigation reported that emodin induces loss of mitochondrial membrane potential and apoptosis through ROS generation in SCC-4 cells. Furthermore, these emodin-dependent apoptotic changes were blocked upon pretreatment with the antioxidant *N*-acetylcysteine [10]. When cells were treated with emodin for various durations, TUNEL assays indicated significant apoptosis in cultures incubated with 10 μM of emodin for 24 h or 20 μM of emodin for 12 h. According to these preliminary results, we incubated IMR-32 cells with various concentrations of emodin for 24 h and analyzed apoptotic signaling. Data from the current study revealed that emodin directly induces oxidative stress in IMR-32 cells (Figure 2). Pretreatment with antioxidants effectively prevented ROS-mediated emodin-induced apoptotic biochemical changes (Figures 2 and 3). ROS is an important upstream regulator of the apoptotic cascade in various mammalian cells [28,29,36]. These earlier results, coupled with our findings, strongly indicate that oxidative injury plays a pivotal role in emodin-induced apoptosis. However, the mechanisms underlying emodin-triggered ROS generation remain to be determined.

The intracellular calcium level is critical in the regulation of cell death [19,37,38]. Previously, we showed that increases in intracellular calcium levels are involved in cell apoptosis triggered by specific chemical compounds [39,40]. Furthermore, intracellular ROS acts as an upstream regulator of the calcium level and induction of apoptosis [39,40]. Recent experiments have disclosed Ca^2+^ concentration changes during emodin-induced cell apoptosis of SCC-4 cells [10]. In the current investigation, we examined whether emodin-induced apoptosis is mediated via intracellular calcium increase in IMR-32 cells. Notably, the [Ca^2+^]i level was increased following emodin treatment, which was largely attributed to release of internal Ca^2+^ from storage organelles (Figure 3A,B). This increase was significantly blocked by NAC (Figure 3B), further indicating that ROS generation is responsible for the increased intracellular calcium concentration in IMR-32 cells.

NO, an endogenous product of NADPH, O_2_ and l-arginine catalysis by nitric oxide synthase, is involved in apoptosis triggered by several types of stimuli [19,20,39,40]. The regulatory actions of NO on mitochondrial apoptotic signaling pathways are well documented. Earlier studies by our group have shown that tamoxifen and co-treatment with methylglyoxal and high glucose promote the intramitochondrial Ca^2+^ concentration, leading to stimulation of mitochondrial NO synthase activity and NO production in rat livers, human breast cancer MCF-7, and human mononuclear cells [20,39,40]. Three NOS isoforms have been identified and associated with various specific tissues and cell types: neuronal NOS (nNOS), Ca^2+^/calmodulin-independent and inducible NOS (iNOS), and endothelial NOS (eNOS).

A previous study also found that emodin could down-regulate the expression of NF-κB and NF-κB-regulated proteins, such as eNOS, and could reduce eNOS phosphorylation in orthotopic pancreatic cancer tissues [41]. eNOS stimulates the release of NO, promoting epithelial cell proliferation [42]. Emodin inhibition of eNOS expression in orthotopic pancreatic cancer tissues may be involved in the inhibition of tumor angiogenesis [41]. Although preliminary experiments have shown that emodin could significantly activate iNOS in IMR-32 cells (data not shown), these data are somewhat ambiguous and require further investigation, including analyses of the upstream regulators of iNOS activation in emodin-treated IMR-32 cells. Experiments from the current study revealed ~3.7-fold higher intracellular NO levels following emodin treatment of IMR-32 cells *versus* untreated controls (Figure 3C). Moreover, pretreatment with EGTA substantially prevented this increase in intracellular NO (Figure 3C), indicating that NO production in emodin-treated IMR-32 cells is dependent on the intracellular calcium concentration. The regulatory role of NO in apoptosis is complex, and NO-mediated apoptotic effects are modulated via different mechanisms in various cell types [18,39,40,43]. For instance, NOS substrates or NO donors inhibit apoptosis induced by photodynamic treatment in CCRF-CEM cells [44]. In addition, a NOS inhibitor suppresses the high glucose and methylglyoxal-stimulated mitochondria-dependent apoptotic pathway [40]. Importantly, PTIO attenuated loss of mitochondrial membrane potential (MMP) and suppressed caspase activation (Figure 4) in our experiments, suggesting that NO is an important mediator of apoptosis in emodin-treated IMR 32 cells.

NO-mediated apoptotic processes are associated with p53 gene activation, which is essential for regulation of the cell cycle and/or apoptotic signaling occurring through p21^Waf1/Cip1^ or Bax [31,32]. In a previous study, we reported apoptotic signaling pathway-induced increases in p53 and p21 levels in human mononuclear cells following co-treatment with high glucose and methylglyoxal [40]. Here, we observed upregulation of both p53 and p21 mRNA following treatment with emodin, which was blocked upon pretreatment with PTIO and NAC (Figure 5). The p53 protein, an expression product of the *p53* tumor suppressor gene, prevents the proliferation of cells with damaged DNA. Damaged DNA triggers ATM kinase activity, which catalyzes phosphorylation of p53 proteins. Accumulation of phosphorylated p53 proteins in DNA-damaged cells activates two types of events, specifically, cell cycle arrest and apoptosis. The p53 protein is a transcription factor that activates specific genes, including *p21*. The p21 protein, a member of the Cdk inhibitor family, blocks activation of the Cdk-cyclin complex, leading to cell cycle arrest. Additionally, the protein activates gene encoding proteins that trigger cell apoptotic processes through binding and inactivation of Bcl2, an apoptotic inhibitor. Our data showed that emodin induces p53 expression (Figure 5A). Our results clearly showed that siRNA-mediated knockdown of p53 significantly decreased the mRNA level of p21 in emodin-treated IMR-32 cells (Figure 6A). In addition, inhibition of p53 and p21 protein expression was accompanied by marked suppression of emodin-induced apoptosis (Figure 6B). These results emphasize that p53 and p21 play important roles in the emodin-induced apoptosis of IMR-32 cells. We hypothesize that following emodin treatment of IMR-32 cells, ROS is generated, which triggers DNA damage, subsequently activating p53 and downstream apoptotic signal cascades.

## 4. Experimental Section

### 4.1. Chemicals and Reagents

Emodin, 3-(4,5-dimethylthiazol-2-yl)-2,5-diphenyltetrazolium bromide (MTT), Dulbecco’s modified Eagle’s medium (DMEM), sodium pyruvate, 2,7-dichlorofluorescin diacetate (DCF-DA), dihydrorhodamine 123 (DHR 123), 2-phenyl-4,4,5,5-tetramethylimidazoline-1-oxyl-3-oxide (PTIO), *N*-acetyl cysteine (NAC), 2,7-dichlorofluorescein diacetate (DCF-DA), propidium iodide, Hoechst 33342 and BAPTA-AM were purchased from Sigma (St. Louis, MO, USA). Anti-p53, anti-caspase-3 and anti-β-actin antibodies were from Santa Cruz Biotechnology (Santa Cruz, CA, USA). Bicinchoninic acid (BCA) protein assay reagent was obtained from Pierce (Rockford, IL, USA).

### 4.2. Cell Culture and Emodin Treatment

Human neuroblastoma IMR-32 cells were cultured at 37 °C in a humid 95% air/5% CO_2_ atmosphere in 90% minimum essential medium (Biochrom KG, Berlin, Germany) supplemented with 10% heat-inactivated fetal bovine serum, 100 IU/mL penicillin, and 100 μg/mL streptomycin. Cells (~5–6 × 10^6^) were incubated in medium containing various concentrations of emodin for 24 h, and cells were then washed twice with ice-cold PBS and lysed on ice for 10 min in 400 μL lysis buffer (20 mM Tris-HCl, pH 7.4, 1 mM EDTA, 1 mM EGTA, 1% Triton X-100, 1 mM benzamidine, 1 mM phenylmethylsulfonyl fluoride, 50 mM NaF, 20 μM sodium pyrophosphate and 1 mM sodium orthovanadate). Cell lysates were sonicated on ice for 3 × 10 s followed by centrifugation at 15,000 × *g* for 20 min at 4 °C. The supernatants were used as cell extracts.

### 4.3. MTT Assay

Cell survival was monitored using the MTT (3-[4,5-dimethylthiazol-2-yl]-2,5-diphenyltetrazolium bromide) test. Briefly, cells were treated with the indicated concentrations of emodin for 24 h, and then treated with 100 μL of 0.45 g/L MTT solution. The cells were incubated at 37 °C for 60 min to allow color development, and then 100 μL of 20% SDS in DMF:H_2_O (1:1) solution was added to each well to stop the reaction. The plates were incubated overnight at 37 °C for solubilization of the formazan products, and spectrophotometric data were measured using an ELISA reader at a wavelength of 570 nm.

### 4.4. Assessment of Necrosis and Apoptosis

Oligonucleosomal DNA fragmentation (a hallmark of apoptosis) was measured using the Cell Death Detection ELISA^plus^ kit (Roche Molecular Biochemicals, Mannheim, Germany). Cells (1 × 10^5^) were treated with or without the indicated concentrations of emodin at 37 °C for 24 h, the procedures were performed according to the manufacturer’s protocol, and spectrophotometric data were obtained using an ELISA reader at 405 nm. In addition, cells were incubated with propidium iodide (1 μg/mL) and Hoechst 33342 (2 μg/mL) at room temperature for 10 min, and fluorescent microscopy was used to identify the percentage of propidium iodide-impermeable cells having condensed/fragmented nuclei (apoptotic) and the percentage of propidium iodide-permeable cells (necrotic). In each experiment, 8–10 independent fields (~500–800 nuclei in total) were counted per condition. The activity of lactate dehydrogenase (LDH) present in the culture medium was evaluated as an additional index of necrosis, as previously described [45–47]. Briefly, cells (5 × 10^4^) were cultured in 96-well microtiter plates (100 μL medium/well), LDH activity was assayed using, and the absorption values at 490 nm were determined with an ELISA reader, according to the manufacturer’s instructions (Promega, Madison, WI, USA). Blanks consisted of test substances added to cell-free medium.

### 4.5. ROS Assay

ROS were measured in arbitrary units using 2,7-dichlorofluorescein diacetate (DCF-DA) or dihydrorhodamine 123 (DHR 123) dye. Cells (1.0 × 10^6^) were incubated in 50 μL PBS containing 20 μM DCF-DA or DHR123 for 1 h at 37 °C, and relative ROS units were determined using a fluorescence ELISA reader (excitation 485 nm, emission 530 nm). An aliquot of the cell suspension was lysed, the protein concentration was determined, and the results were expressed as arbitrary absorbance units/mg protein.

### 4.6. Detection of Intracellular Calcium Concentration ([Ca^2+^]i )

The [Ca^2+^]i was detected with Fluo-3 AM fluorescence dye, using a modification of the previously reported method [19,48]. Briefly, cells were co-treated with emodin, harvested and washed, and then loaded with 6 μM Fluo-3 AM in standard medium (140 mM NaCl, 5 mM KCl, 1 mM MgCl_2_, 5.6 mM glucose, 1.5 mM CaCl_2_, and 20 mM Hepes, pH of 7.4). After 30 min, the cells were washed 3 times with PBS and then resuspended in standard medium or Ca^2+^-free standard medium. The fluorescence intensity of Fluo-3 was determined using a fluorescence spectrophotometer (Hitachi, F-2000, Tokyo, Japan, excitation at 490 nm, emission at 526 nm).

### 4.7. Detection of Intracellular NO Content

The DAF-2DA fluorescence dye was used to detect intracellular NO, according to a modification of the previously reported method [19,49]. Briefly, treated or control cells were collected and washed, and then incubated with 3 μM DAF-2DA. After 60 min, the cells were washed 3 times with PBS and the fluorescence intensity was measured by a fluorescence spectrophotometer (Hitachi, F-2000; excitation at 485 nm, emission at 515 nm).

### 4.8. Caspase Activity Assays

Caspase-9 activity was assayed using the Colorimetric Caspase-9 Assay Kit (Calbiochem, CA, USA). Caspase-3 activity was measured using the Z-DEVD-AFC fluorogenic substrate, as previously described [36,50].

### 4.9. Real-Time RT-PCR Assay

Total RNA was extracted with the TRIzol reagent (Life Technologies, Paisley, UK ) and purified with an RNeasy Mini kit (Qiagen, Valencia, CA, USA ), according to the manufacturers’ protocols. Real-time PCR was carried out with an ABI 7000 Prism Sequence Detection System (Applied Biosystems, Foster City, CA, USA ). The β-actin mRNA levels were quantified as an endogenous control, and used for normalization. The primers used for PCR were as follows: p53, 5′-CCC ATC CTC ACC ATC ATC AC-3′ and 5′-GTC AGT GGG GAA CAA GAA GTG-3′; p21, 5′-GCC GAA GTC AGT TCC TTG TGG A-3′ and 5′-GTG GGC GGA TTA GGG CTT-3′.

### 4.10. siRNA Knockdown

Lipofectamine was used to transfect mononuclear cells with 150 nM of siRNA for targeting against p53 (5′-GACUCCAGUGGUAAUCUACTT-3′; sip53) or a scrambled control duplex (5′-GCGCGCUUUGUAGGAUUCG-3′; siScr). Twenty-four hours post-transfection, fresh culture medium was added, and the cells were treated with or without 20 μM emodin for another 24 h.

### 4.11. Immunoblots

Proteins were transferred from SDS-PAGE gels to PVDF membranes, and probed with antibodies against p53 or active caspase-3. Immunoreactive bands were detected using an alkaline phosphatase-conjugated goat anti-rabbit IgG antibody and visualized using the CDP-Star™ kit, according to the manufacturer’s protocol (Mannheim, Germany).

### 4.12. Statistics

Data were analyzed using one-way ANOVA, and differences were evaluated using a two-tailed Student’s t-test and analysis of variance. *p* < 0.05 was considered significant.

## 5. Conclusions

**Figure 7:**
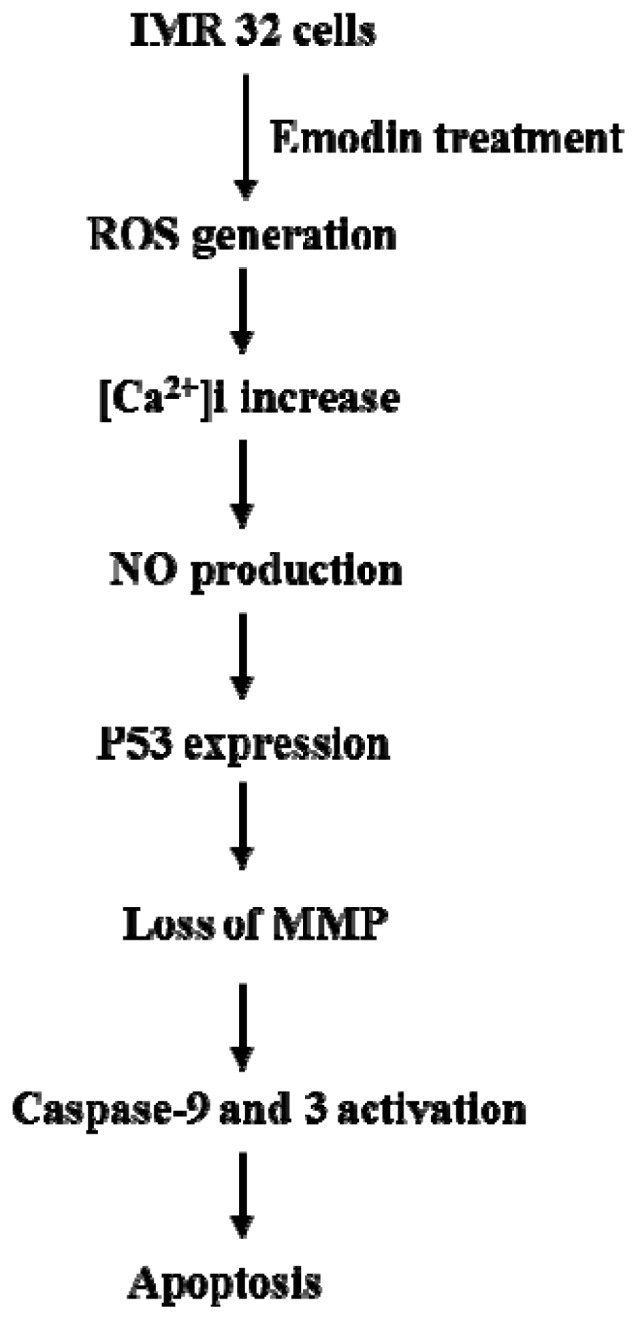
Summary of events occurring during emodin-induced apoptosis in IMR32 cells.

Our results suggest that emodin directly promotes ROS generation, which, in turn, triggers calcium influx from intracellular Ca^2+^ storage organelles, leading to increased intracellular calcium concentrations that stimulate NO production. Moreover, pretreatment with NAC or PTIO blocks emodin-induced upregulation of critical genes and rescues cell viability. Based on these findings, we propose that intracellular ROS and NO play critical roles in emodin-induced apoptosis of IMR-32 cells and propose a signaling regulatory model in Figure 7.
